# User-Agent Bond in Generalizable Environments: Long-Term Risk-Reduction via Nudged Virtual Choices

**DOI:** 10.3389/fpsyg.2021.695389

**Published:** 2021-08-26

**Authors:** Liyuan Wang, John L. Christensen, Benjamin J. Smith, Traci K. Gillig, David C. Jeong, Mingxuan Liu, Paul R. Appleby, Stephen J. Read, Lynn C. Miller

**Affiliations:** ^1^Department of Communication, Annenberg School for Communication and Journalism, University of Southern California, Los Angeles, CA, United States; ^2^Department of Communication, University of Connecticut, Storrs, CT, United States; ^3^Department of Psychology, University of Oregon, Eugene, OR, United States; ^4^Department of Strategic Communication, Edward R. Murrow College of Communication, Washington State University, Pullman, WA, United States; ^5^Department of Communication, Santa Clara University, Santa Clara, CA, United States; ^6^Department of Psychology, University of Southern California, Los Angeles, CA, United States

**Keywords:** agent (virtual users), generalizable virtual environments, identification, intervention, risk-reduction behavior, men who had sex with men, virtual choices, video game

## Abstract

Avatars or agents are digitized self-representations of a player in mediated environments. While using agents to navigate through mediated environments, players form bonds with their self-agents or characters, a process referred to as identification. Identification can involve automatic, but temporary, self-concept “shifts in implicit self-perceptions” (Klimmt et al., 2010, p. 323) of the media user by adopting or emphasizing the action choices on behalf of the social expectation of the avatar in the mediated environment. In the current study, we test the possibility that users' identification with video game avatars–a bond built between avatars and players- would account for subsequent behavior changes. We did so by using 3-month longitudinal data involving a narratively-based serious game: Socially Optimized Learning in Virtual Environments (SOLVE), a 3D-interactive game designed to reduce risky sexual behaviors among young men who have sex with men (*n* = 444). Results show that video game identification predicts both the reduction of risky sexual behaviors over time, and reduction in the number of non-primary partners with whom risky sex occurs. And when players identify with the game character, they tend to make healthier choices, which significantly mediates the link between video game identification and reduction of risky behaviors.

## Introduction

Avatars^1^, or self-agents, are digital self-representations of a player in mediated environments. Players can form bonds with their self-agents or characters, a process referred to as identification (Klimmt et al., 2010). Identification can involve automatic, but temporary, self-concept “shifts in implicit self-perceptions” (Klimmt et al., 2010, p. 323) of the media user by adopting or emphasizing the type of action choices (e.g., racing, military) of the user's character—given the options available in-context. Identification can be enhanced in a variety of ways, for example, by affording players opportunities to customize their characters (Turkay and Kinzer, 2014) or by enhancing the user's sense of presence (Christy and Fox, 2016)^2^. Furthermore, across a range of experimental studies, meta-analysis indicates that players appear to adjust their attitudes and behaviors to be more in-line with the presumed, mostly physically-based characteristics (e.g., size, gender), of their avatar (Ratan et al., 2020). Together, these findings suggest that for the avatar with whom one identifies, the avatar's situated behaviors, as well as their appearance (e.g., similarity to self), may produce change that might be longer lasting. For example, Birk and Manyk (2019) manipulated customization (or not) of avatars and exposure to an attentional retraining therapy to reduce anxiety. Participants in the training condition, but only those who could customize their avatars, experienced reduced anxiety, although the duration of these effects was unclear.

There has long been interest in using virtual environments (VE) for longer-term behavior change, including for changing health behaviors (e.g., Read et al., 2006; Baranowski et al., 2008). There are numerous reviews of the role of games or virtual environments (Papastergiou, 2009; Lu et al., 2012a,b; Bleakley et al., 2015) in producing behavior change. However, there are relatively few studies examining the role of avatars in health outcomes. One study indicates that avatar choices (e.g., relevant to alcohol or water choice) predict 3-month alcohol use (Wang et al., 2018). The results suggested that avatar choices can have long-term effects in everyday life, but did not include an examination of identification as a factor. There has been no published work, of which we are aware, examining the relationship between the strength of human-avatar bonds (e.g., identification) and longer-term behavior change (e.g., over 3 months), including in the health domain.

In the current study, we proposed and tested whether user's identification with video game avatars (Hefner et al., 2007; Klimmt et al., 2010), indicative of a special bond built between avatars and players, would predict subsequent behavior changes. We did so by using 3-month longitudinal data involving a narratively-based serious game developed by our research team: Socially Optimized Learning in Virtual Environments using Intelligent Technologies (SOLVE-IT) (Miller et al., 2011; Christensen et al., 2013) is a 3D-interactive game designed to reduce risky sexual behaviors among young men who (YMSM) have sex with men^3^.

### Identity-Bond and Change: Generalizable Virtual Environments

Serious games (Sawyer and Smith, 2008), also known as persuasive games (Bogost, 2007) or game-based learning (Connolly et al., 2012), usually refer to video games that are delivered via VE with the goal of promoting education, training, and behavioral changes (Connolly et al., 2012). In the past, those “games” have long been associated with repetitive drills and simulations. Now, with advances in media technology, an increasing number of serious games are engaging and entertaining, especially those with interactive and narrative elements enabling users to choose their own adventures (Green and Jenkins, 2014) and to be transported to the virtual world (Green and Brock, 2000)” of their avatars' or game characters' (Baranowski et al., 2008). A single team reviewed serious games, first from the earliest up to 2012 (Connolly et al., 2012) and then from 2012 to 2016 (Boyle et al., 2016): In the latter review, four times as many games (20)–incorporating story-lines or narrative structures such as action, adventure, or role-play—were identified. Indeed, through incorporating narrative structures, serious games have morphed and evolved into not just effective, but also fun and enjoyable, interventions (Lu et al., 2012a).

But as far as we know, narrative structures in serious games are often not specifically designed to have generalizability to everyday life (GEL; see Miller et al., 2019a)^4^. That is, while serious games are designed to change people's behaviors/decision making and attitudes, rarely are these serious games designed using representative sampling of the everyday life environments to which one wishes to generalize^5^. Indeed, we probably only need small puzzle games like Fruit Saga to train students to understand math. But, where we are changing—what are sometimes automatic social responses in context—deeply understanding and representing those situations, sequences, etc. and what is emotionally happening in them (in order to change behavior and decision-making in similar contexts in everyday life) is another matter. It is important that participants are “trained” and “practiced” in a virtual environment that is representative of the challenging scenario participants face in everyday life. Indeed, GEL matters because we want those learned response patterns in the virtual environment in response to a situation to transfer to similar situations in those individuals' everyday life (Miller et al., 2019a,b).

Building GEL into virtual environment implementations, as described above, relies on interactive narrative structures. Interactive narratives (IN) in virtual environments are systems affording the user the ability to assume the role of a character in a story (narrative) as that user interacts with other character agents. IN can afford interventions using virtual environments (for brief review see Miller et al., 2019a) by providing the critical cues (e.g., visual, acoustic, etc.) through which users re-imagine and experience trauma triggering events with a trained therapist (see Rizzo et al., 2014; Rizzo and Koenig, 2017) or just work through challenging situations with intelligent agent mentors/guides (e.g., Marsella et al., 2000; Christensen et al., 2013). In reviews of serious games (Connolly et al., 2012; Boyle et al., 2016), an interactive narrative structure (i.e., involving action, adventure, or role-play) was frequently identified. Often, serious games with narrative structures have been found to be not only effective, but fun and enjoyable, interventions (Lu et al., 2012a). This is the case even if the behaviors of interest for their persons of interest in these serious games might not be so “enjoyable,” such as eating more fruit and vegetables for children (Baranowski et al., 2003), glucose management for diabetics (Thompson et al., 2010), and reduction of risky sexual behaviors for YMSM in SOLVE (Christensen et al., 2013).

In designing SOLVE (Read et al., 2006), the goal was to place YMSM in an IN virtual environment designed to simulate the emotional, interpersonal and contextual narrative of actual sexual encounters while challenging (with guides) and changing MSM's more automatic patterns of their responses. The SOLVE-IT (SOLVE with intelligent technologies) intervention involves an animated interactive virtual dating narrative game in which users choose their characters' skin coloring, hair color, and make clothing choices for their characters that are then aged to create a “virtual future self (VFS)” that, like SOLVE guides, scaffolds the user in making a series of risky or safe choices in interacting with potential sexual partners, so that “over time, self-regulated learners actively instruct and reinforce themselves, gaining confidence and self-efficacy in their ability to understand and succeed in achieving their goals, while avoiding social and physical harm (Read et al., 2006, p. 2).” The VFS's role as an intervention agent is to optimize the user's self-regulated learning. The VSF does so like a good parent (Nelson, 1993) through interactivity, scaffolding, and VSF (coach) responsiveness, and reframing the ongoing narrative leading up to decisions that may be risky in every component of the process outlined below. These components are theoretically grounded in what we refer to as the *recursive narrative regulatory circuitry* that depends upon the active interaction of the user and socially facilitated processes (Read et al., 2006) and include: (1) encouraging the user to read his and the other's affective cues (e.g., attraction and desire for this man); (2) help user interpret and make inferences for the self and other's affective cues (what they mean- cause, effect, intent); (3) encourage user to clarify their own and the other's interpersonal goals; (4) generate possible solutions: develop plans to optimize goals, taking resources into account; (5) help them weigh and make a decision (6) guide them in acting on the decision to enact behavior. SOLVE was one of the first behavioral interventions to make emotions (and acknowledging them) more central (instead of secondary at best) in the ongoing narrative decision-making process. Thus, the VFS guide provided a kind of “glue” scaffolding and reframing the situation and linking and moving cognitive and affective reactions to promote behavior change (Read et al., 2006) while linking past, current, and future decisions.

In an IN structure, the bonds that form between humans and their avatars (or agents)—that represent them digitally—can be strong. The strength of those bonds can predict an array of outcomes, including positive health effects (Kim and Sundar, 2012; Birk et al., 2016). Avatars (or agents) are virtual digital representations through which a user (i.e., player in a digital game or virtual environment) assumes the role of a character. Nowak and Fox (2018) define avatars as “*digital representations that symbolizes the self in the interaction”* (p. 30). Agents are used in a similar fashion, except that agents can also broadly refer to animate objects (including humans or animals) as well as inanimate objects. Here, by agents we refer to digital representations that either symbolize the *self or others* in the social interaction. These human-avatar (or human/self-agent) bonds have been referred to as “identification” (i.e., Klimmit et al., 2009). Klimmit et al. (2009) define video game identification as “a temporary alteration of media users' self-concept through adoption of perceived character of a media person” (p. 258). Indeed, successfully navigating one's ideal future self in a dating scene could make one form strong bonds with one's own avatar. There is considerable evidence that these bonds are often quite strong. While reviews have shown that increasing game addiction was related to the strong identification bonds between gamers and their avatars (Lemenager et al., 2020), the strength of these bonds could also be used in achieving healthy behavioral changes in serious games (Papastergiou, 2009; Primack et al., 2012; Bleakley et al., 2015).

Therefore, we specify the hypothesis below.

*H*_1_: Identification with one's virtual future self game character is positively associated with subsequent real-life reduction of risky behavior among YMSM who play SOLVE, an IN game designed to enhance safer sexual practices.

### How Does Identification Affect Virtual Choice/Behaviors?

The conceptualization of video game identification can also speak to another important aspect of serious games: *virtual choices*. Virtual choices are part of the interactivity features that are offered in video games with the purpose of enhancing media richness such that users may be more likely to explore the mediated interface (Sundar et al., 2013). When offered in video games to entertain, virtual choices may simply be interactive features that only garner players' attention (e.g., “should my avatar take the path to the south,” or “should he be wearing a blue or a red shirt today”). However, when offered in serious video games that are created to be representative of real-life situations, virtual choices have applied implications. They are assessment tools of performances or behaviors that are otherwise hard-to-observe in real life situations (Rizzo et al., 2000; Blascovich et al., 2002). Virtual performances such as scientific inquiry abilities (Ketelhut et al., 2010) and surgical skills (Seymour et al., 2002; Grantcharov et al., 2003) in serious games have been used to assess the (likely) subsequent real-life performance of targeted participants. This assessment potential of virtual choices is also applicable to serious games interventions that target real-life challenges. In those cases, participants' “virtual choices themselves may indicate current movement in behavior toward key intervention messages provided by a game character (e.g., a scaffolding mentor or guide) and thus be prognostic of future behavior change” (Wang et al., 2018, p. 4).

#### Nudged Virtual Choices in Line With Ideal Self or Intervention Goal

Thaler and Sunstein (2009) define a “nudge” as “any aspect of the choice architecture that alters people's behavior in a predictable way without forbidding any options or significantly changing their economic incentives. To count as a mere nudge, the intervention must be easy and cheap to avoid” (p. 6). Participants of the SOLVE-IT game, for example, while walking through a series of dating scenarios, may find themselves at different choice points where they need to make *virtual choices*. Their virtual choice to drink more water in a house party, for example, meets “nudge” criteria: It is a cheap and easy to avoid option (the alternative is to drink alcohol), and indicates their compliance with the persuasive messages embedded in the game. Indeed, Wang et al. (2018) reported significant correlations between virtual choices of non-alcoholic beverages and reductions of actual alcohol intake post SOLVE intervention.

The importance of video game identification can also be evaluated by its associations with virtual choices such that serious games with greater potential in eliciting identification are more likely to induce more desirable in-game virtual behaviors. To be specific, the richness of interactive features that are offered by video games, players' “temporary alteration of self-concept” may enable them to act upon the perspectives of the game character. As Klimmit et al. (2009) suggested, players of a first-person shooting game who are subconsciously integrating the identity of a soldier would perceive themselves as being “more courageous, stressed, cautious, aggressive, violent, dutiful, etc., than they would be under “normal” circumstances” (2009, p. 358). Since they are “soldiers” in the game, they would also be acting more courageous, vigilantly, cautiously, and aggressively in shooting, sniping, or flanking the enemy in the game. Similarly, players can be influenced by an ideal virtual future self (i.e., VFS mentor that is like one's self character but “aged” and wiser in making safer sex choices) that is designed in serious games like SOLVE-IT. Given the VFS, participants may alter their virtual self-concepts in line with the VFS, therefore making healthier virtual choices based on their avatar's desired identity. They may choose to act, virtually, in more responsible ways such as choosing a safer way of sex or reducing their beer intake because they are identified with their ideal version of themselves [in a game designed to “*nudge*” (Thaler and Sunstein, 2009) individuals toward safer choices]. Therefore, we can expect significant associations between video game identification with the VFS and virtual choices, which could also subsequently predict behavior changes, or the efficacy of serious game interventions.

*H*_2_: Virtual choices mediate the relationship between video game identification with the virtual future self (VFS) mentor and subsequent behavior change.

## Method

### Study Design and Participants

Study design and participant's data for this analysis were drawn from the SOLVE-IT intervention. In the original design, YMSM were randomly assigned to the game or a waitlist control condition (*N* = 934). As video game identification could only be accessed for those who played the game, our analysis focused on just those in the experimental condition (*N* = 444). Those participants have a mean age of 22.13, with 76.1% identified as white/Caucasian, 12.4% as Latino/Hispanic, and 11.5% as Black/African American. Recruitment criteria and more detailed social economic backgrounds are also described elsewhere (see Christensen et al., 2013; Wang et al., 2018) and, as relevant, below.

“*Participants were recruited nationwide in the United States through banner advertisements placed on Craigslist, blogs, and gay interest websites. Criterion for inclusion were (1) receiving a prior HIV-negative test result, (2) living in the United States, (3) being 18–24 years of age, and (4) engaging in CAI* [condomless anal intercourse] *with a non-primary male partner during the three-month period prior to enrollment in the study. “Non-primary partner” refers to a male partner with whom the participant was not, at the time, engaged in a romantic relationship (for additional details see Christensen et al.*, *2013). This study was approved by the university's institutional review board (IRB). Participants were only identified by email address to enhance confidentiality. Email addresses were subsequently deleted upon study completion (Wang et al., 2017, p. 15).”*

Each participant played the SOLVE-IT game at least once during the intervention, though a few were shown to have played multiple times. Participants began their game by customizing their avatar (customizing hair color, skin color, eye color, clothes, shoes, etc.) who was then “aged” for all players to become the user's VFS.

### Measures

#### Risky Behaviors

Unsafe sexual behaviors in real life were operationalized in two ways (i.e., within a period of time, numbers of non-primary partners with whom had unsafe sex; total count of unsafe sex). First, we examined the number of Non-Primary Partners with whom participants had condomless anal intercourse (CAI). This was a composite measure that added the number of partners with whom participants had condomless receptive anal sex and those with whom they had condomless insertive anal sex. At baseline, participants reported the number of non-primary partners with whom they had engaged in CAI in the past 90 days. Participants gave a report of the same measure at a 3-month follow-up, and this afforded a measure of behavior change. Second, we assessed the actual count of CAI incidents in the past 90 days (a composite of responses for insertive CAI and receptive CAI) at baseline and 3-month follow-up affording a second measure of behavior change. Virtual Safe-Sex Choices.

**Figure 1:**
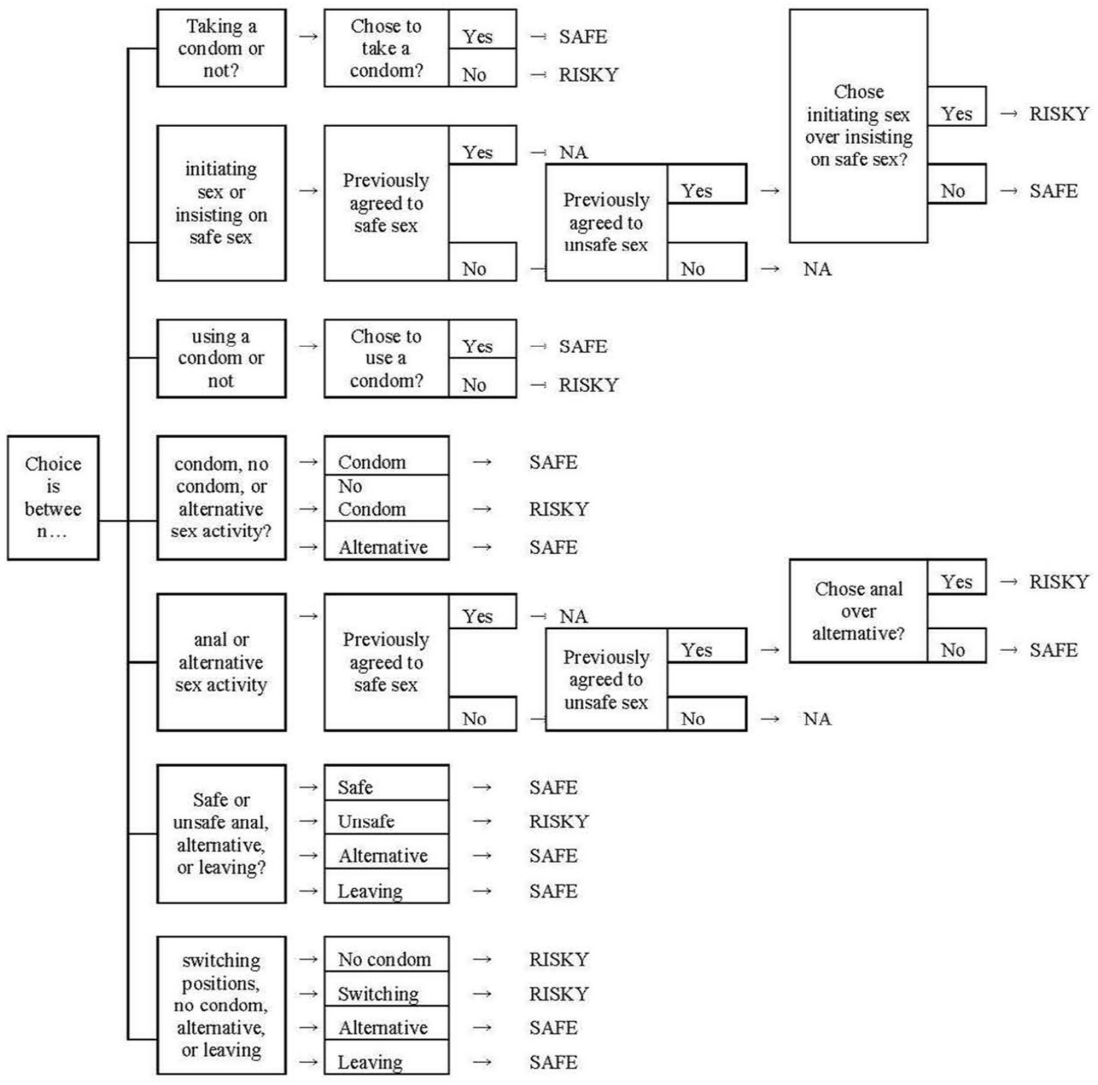
Risk choice and safe sex procedures. This figure shows the procedures used to determine whether a sexually risky decision choice is safe or risky in the SOLVE game by taking into account the context of the choice. “NA” indicates a particular choice is not relevant to sexual risk-taking at all. This figure also appeared in the work by Smith et al. (2018). Authors are licensed to use this material, License number: 5047310486925.

Virtual sexual behaviors were operationalized in terms of the number of choices made by the participants during two levels of game play. Decisions were categorized and recorded based on their consequences in leading specifically to either safe or risky sexual intercourse (see Figure 1). Participants made from 0 to 6 safe choices, with higher values indicating more safe choices.

#### Video Game Identification

Three items were created to evaluate video identification with the Virtual Future Self (VFS) character to fit with the SOLVE design. Sample items included, “I could identify with the Virtual Future Self character,” “The virtual future-self character was trustworthy,” and “The virtual future-self character was believable.” Participants rated game involvement items on a 1 (strongly disagree) to 10 (strongly agree) scale (α = 0.92).

## Result

### Preliminary Data Analysis

Following Christensen et al. (2013), we used residualized change scores in order to eliminate dependency between simple difference scores and baseline values (MacKinnon, 2012; Muthen and Muthen, 2012) and potential problems with reliability of measurement (Rogosa et al., 1982; Tisak and Smith, 1994; McFarland and Ryan, 2000; Edwards, 2001). Higher scores on variables indicate more game identification, safer choices, more CAI and numbers of partners over time. Table 1 shows the bivariate correlations of each variable of interest. As we have hypothesized, video game identification was significantly correlated to all our outcomes of interests.

**Table 1:** Bivariate correlations of variables of interests.

	**1**	**2**	**3**	**4**	***M***	***SD***
1. Video game identification	1	-	-	-	7.66	1.86
2. Virtual choice	0.45^**^	1	-	-	5.56	3.07
3. Changes in non-primary Partners to have CAI over 3 months	−0.23^**^	−0.20^**^	1	-	0.16	8.77
4. Changes in CAI over 3 months	−0.35^**^	−0.26^**^	0.80^**^	1	0.1	6.65

** *Correlation is significant at the 0.01 level (2-tailed)*.

*Higher scores for mean more identification, more virtual safer choices, and more partners and more unprotected coondomless anal sex over 3 months. Therefore, negative correlations reflect the role of 1 and 2 in reducing CAI and partners over time*.

### Main Analyses

**Figure 2:**
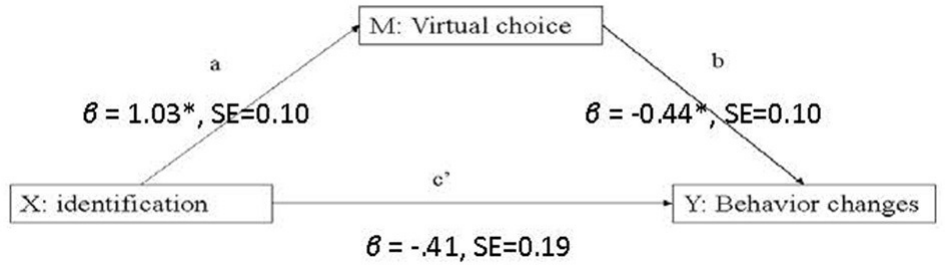
Statistical model for analysis. The current figure shows the Hayes mediation model, and an example of how X: identification affects M: Virtual Choices and Y: Behavior Changes (i.e., change of Non-primary CAI partners). *p < 0.05.

To test H_1_ and H_2_, we used ordinary least squares (OLS) regression provided by the SPSS macro PROCESS (Preacher and Hayes, 2008; Hayes, 2009) using model 4, shown in Figure 2 (here, simple mediation with one mediator per outcome). Table 2 shows the detailed results for each hypothesis. H_1_ posits that video game identification contributes to behavior change in serious games over 3 months. This hypothesis is supported. Identification significantly reduced the amount of CAI, β = −0.56, SE = 0.29, 95% CI: (−1.14, −0.01), and reduced the number of non-primary partners with whom CAI happened: β = −0.41, SE = 0.19, 95% CI: (−0.77, −0.03). H_2_ posits that virtual choices mediate the relationship between identification and behavioral change. This is supported as well. We saw an indirect effect of virtual choices mediating the relationship between identification and changes of CAI, β = −0.48, SE = 0.13, 95% CI: (−0.74, −0.24), and this mediation model explained a significant proportion of variance in the reduction of non-primary CAI partners, *R*^2^ = 0.20, *p* < 0.001. Similarly, we saw an indirect effect of virtual choices mediating the relationship between identification and change of CAI partner counts, β = −0.45, SE = 0.11, 95% CI: (−0.69, −0.25). This mediation model explained a significant proportion of variance in the reduction of non-primary CAI partners, *R*^2^ = 0.18, *p* < 0.001.

**Table 2:** Path coefficients, SE, and 95% CI.

			**95% CI**	
	**Coeff**.	**SE**	**Upper**	**Lower**
**Y: CAI changes**				
a	0.76	0.09	0.59	0.93
b	−0.63	0.05	−0.97	−0.3
c'	−0.56	0.29	−1.14	−0.01
*Indirect effect of virtual choice*				
	−0.48	0.13	−1.14	−0.01
**Y: Changes in non-primary CAI partners**				
a	1.03^*^	0.1	0.84	1.23
b	−0.44^**^	0.1	−0.64	−0.24
c'	−0.41	0.19	−0.77	−0.03
**Indirect effect of virtual choice**				
	−0.45	0.11	−0.69	−0.25

*a, b, and c' corresponds to the paths legend in Figure 2, X: Identification, M: Safe choices, Y: real-life behavior changes (CAI and changes in Non-primary CAI partners)*.

* *p < 0.05*,

** *p < 0.01*.

## Discussion

Results of this study were consistent with previous studies regarding the importance of video game identification (Lu et al., 2012b) in demonstrating positive behavioral change. We found that game virtual future self (VFS) identification significantly related to the reduction of risky behaviors. Our results have shown that video game identification predicted not only the reduction of CAI, but the number of non-primary partners with whom CAI occurs. Results of this study call for closer attention in designing more engaging serious games, and perhaps including VFS, with which and whom players can identify. In subsequent studies, it is therefore important to further consider various means to enhance video game identification to create more effective interventions for behavior change.

This study also found support for the role of video identification's influence on virtual choices. As our results have shown, when players identify with the VFS game character, they tend to make more desirable (healthier) choices. Indeed, a representatively designed real-life-based serious game can not only serve as a means of observation, but could also be utilized as a means of creating behavioral change to facilitate safer sex. With multiple interventions reporting the effectiveness of serious game interventions and targeted campaigns, including SOLVE-IT, on reducing risky behaviors (especially risky sexual behaviors) of YMSM, enhancing video game identification in health settings creates the potential of creating more specialized communication-based interventions.

### Limitations and Future Directions

While demonstrating the potential diagnostic application of virtual choices, this study has certain limitations. First, we had to rely on participants' self-report of their past risky behaviors and involvement. Indeed, there was a lack of biobehavioral measures (e.g., HIV tests; STD tests) in the SOLVE intervention. However, such measures have been rare in other studies on serious games or in other HIV prevention interventions due to their significant cost, especially in a large national sample. If future work can more rigorously measure risky behavior, it may provide a more accurate assessment of behavioral change. Second, because of the design of SOLVE, which had built-in messages—within the game—to remind participants of the potential risk involved in certain behaviors, we could not manipulate the levels of video game identification and therefore compare the “dosage” of identification. In the future, if researchers can create different versions of serious games to enable such comparison, we can gain more knowledge regarding the best ways to enhance video game identification to create more effective interventions.

## Data Availability Statement

The raw data supporting the conclusions of this article will be made available by the authors, without undue reservation.

## Ethics Statement

The studies involving human participants were reviewed and approved by USC IRB. The ethics committee waived the requirement of written informed consent for participation.

## Author Contributions

LW and LM lead the writing team for this manuscript. LW, LM, JC, DJ, SR, and TG were responsible for an initial draft, research design, and ideas in conceptualizing the analysis of the data. LW, BS, TG, ML, and LM were responsible for research and revisions. JC suggested the VFS initially and JC, PA, LM, and SR developed the idea of the virtual future self (VFS) and identification measures for the VFS for SOLVE-IT used in the current manuscript. PA, JC, SR, and LM contributed to game formative research, systematic representative design, and development and all measurement choice and construction initially. PA managed and orchestrated the overall NIH funded SOLVE-IT project, day-to-day operations, and all data collection. LM, JC, PA, SR, DJ, LW, and TG also contributed more recently to systematic representative design discussions relevant to the current work. LW was responsible for analyzing the data, tables and figures, and in writing up result drafts and changes. JC provided supplementary art from SOLVE-IT. All authors contributed to the article and approved the submitted version.

## Author Disclaimer

The content is solely the responsibility of the authors and does not necessarily represent the official views of the National Institute of Mental Health.

## Conflict of Interest

The authors declare that the research was conducted in the absence of any commercial or financial relationships that could be construed as a potential conflict of interest.

## Publisher's Note

All claims expressed in this article are solely those of the authors and do not necessarily represent those of their affiliated organizations, or those of the publisher, the editors and the reviewers. Any product that may be evaluated in this article, or claim that may be made by its manufacturer, is not guaranteed or endorsed by the publisher.

## Footnotes

^1^Technically, agents are a broader category that includes digital representations for the self as well as digital representations of others (whether human or animate) and even includes objects (Lim and Reeves, 2010).

^2^Social presence has been defined as “a psychological state in which the virtuality of experience is unnoticed (Lee, 2004, p. 494).”

^3^SOLVE-IT builds on theory and methods developed in SOLVE, using interactive video and human actors (Miller and Read, 2005; Read et al., 2006).

^4^The logic of built-in generalizability to everyday life (GEL) is based on sampling theory—including extensive formative research with the target audience and then sampling from the sequences and situations leading up to the risky situations of interest (see Miller et al., 2019a for a detailed description).

^5^In addition to sampling logic, we also conduct virtual validity tests to ensure that patterns of stimulus-responses in the same individuals' everyday life situation are correlated with similar situation-response patterns in the virtual environment (Godoy et al., 2013).
